# Targeted Delivery of Potent Chemical Drugs and RNAi to Drug-Resistant Breast Cancer Using RNA-Nanotechnology and RNA-Ligand Displaying Extracellular vesicles

**DOI:** 10.59566/isrnn.2024.0101016

**Published:** 2024

**Authors:** Yuan Soon Ho, Tzu-Chun Cheng, Peixuan Guo

**Affiliations:** 1 Institute of Biochemistry and Molecular Biology, College of Life Sciences, China Medical University, Taichung, Taiwan;; 2 Division of Pharmaceutics and Pharmacology, College of Pharmacy; Center for RNA Nanotechnology and Nanomedicine; James Comprehensive Cancer Center, College of Medicine, The Ohio State University, Columbus, OH, 43210, USA

**Keywords:** RNA, Breast cancer, Drug resistance, Influx and Efflux Transporters

## Abstract

This review describes a new technology to treat breast-cancer-drug-resistance by targeting the ABC as the multi-homo-subunit ATPase, enlightening by the Christmas-lighting budge with serial circuit and the asymmetrical homo-hexamer of the phi29 DNA packaging motor with sequential revolving mechanism. Chemotherapeutics has been widely used in breast cancer treatments, but drug resistance has raised a serious concern. RNA therapeutics has emerged as the third milestone in pharmaceutical drug development. RNA nanoparticles are dynamic, mild, and deformative, resulting in spontaneous, rapid, and efficient accumulation in tumor vasculature after IV injection. Their negative charge and favorable size bypass the nonspecific targeting of vital organs and normal cells. This motile and deformable nature also led to the fast passing of glomerular filters and their movement into the urine for rapid body clearance for those non-tumor-accumulated nanoparticles, resulting in undetectable toxicity. Extracellular vesicles have shown potential as a delivery system for RNAi and chemotherapeutic drugs *in vivo*, contributing to the efficacy of cancer remission. However, the lack of cell-targeting ligands on extracellular vesicles and the nonspecific entry into healthy cells has led to safety concerns. This review addresses how to apply RNA nanotechnology and RNA-ligand displaying extracellular vesicles for specific delivery to breast cancer. The particular focus is on using and combining the RNA and extracellular vesicle technology to deal with breast cancer drug resistance. The targeting capabilities and drug safety can be improved through extracellular vesicle engineering techniques, such as affixing ligands on the extracellular vesicle surface utilizing arrow-tail RNA nanoparticles, ultimately addressing off-target effects and toxicity. Using RNA ligands for specific targeting and the efficient membrane fusion of extracellular vesicles has enabled the development of ligand-displayed extracellular vesicles capable of delivering both RNAi and chemical drugs to cells with precision, effectively inhibiting tumor growth. The negative charge inherent in the vesicles results in electrostatic repulsion, reducing non-specific binding to healthy cells that contain negatively charged lipid membranes. By leveraging the principles of RNA nanotechnology, the engineering of extracellular vesicles offers a promising avenue for addressing breast cancer drug resistance. This review also discusses applying the series of circuit mechanisms in Christmas-decorating-lighting to develop effective therapeutics to combat breast cancer chemoresistance by targeting the ABC drug transporter and breast cancer surface receptors.

## INTRODUCTION

Breast cancer, forming in breast cells, is the most prevalent cancer in women globally and can affect men. Treatments typically combine surgery, chemotherapy, radiation, hormone, and targeted therapies, but drug resistance often leads to treatment failure and disease progression. Understanding drug resistance is essential for improving patient outcomes and survival. This review examines current knowledge of drug resistance in breast cancer. This review explores the current understanding of drug resistance in breast cancer. It explores strategies to overcome drug resistance and how RNA nanomedicines can facilitate the development of more effective, personalized treatments for patients with drug-resistant breast cancer.

RNA nanoparticles possess exceptional motile and deformable capabilities, making them ideal for advanced therapeutic uses. Utilizing RNA nanotechnology, scientists are creating innovative treatments that can naturally navigate tumor vasculature, target cancer cells, and boast favorable biodistribution for quick renal excretion, reducing toxicity. RNA’s dual role as a therapeutic agent and delivery medium marks it as a promising asset in cancer treatment. Extensive studies have underscored the novel application of RNA nanotechnology in adorning extracellular vesicles with breast cancer ligands to deliver both chemotherapy drugs and RNA interference (RNAi) to tackle breast cancer chemoresistance. Particularly emphasized is the use of the GalNac ligand and miRNA to silence the ABC drug efflux pump, which are critical strategies in overcoming drug resistance.

### The categorization and identification of surface markers in breast cancer.

Breast cancer classification is based on tumor location, receptor presence, and spread extent. The common system categorizes it by the presence of estrogen receptors (ER), progesterone receptors (PR), and human epidermal growth factor receptor 2 (HER2). Additionally, tumor cell appearance under a microscope, like ductal carcinoma in situ (DCIS) or invasive ductal carcinoma, is used for classification. Surface markers such as HER2/neu, ER, and PR further characterize breast cancer and guide treatment decisions. These markers assist oncologists in determining the most effective treatment options, including targeted and hormone therapies, based on the tumor’s specific characteristics.

### An Overview of Existing Systemic Therapies for Breast Cancer Treatment

Currently, there are several drugs available for the systemic treatment of breast cancer (Table 1). These include hormone therapy drugs such as tamoxifen, aromatase inhibitors (e.g., anastrozole, letrozole, exemestane), and selective estrogen receptor degraders (SERDs) like fulvestrant. Additionally, targeted therapy drugs such as trastuzumab, pertuzumab, and adotrastuzumab emtansine are used for HER2-positive breast cancer. Chemotherapy drugs such as paclitaxel, doxorubicin, and cyclophosphamide are also commonly utilized in the treatment of breast cancer^1^.

### Mechanisms of Chemoresistance and Multidrug Resistance Within Breast Cancer

Chemoresistance and multidrug resistance^2^ are complex mechanisms that contribute to the challenge of treating breast cancer. These mechanisms can arise from various factors, including genetic mutations, tumor heterogeneity, and the tumor microenvironment. One common mechanism of chemoresistance is the upregulation of drug efflux pumps, such as P-glycoprotein, which actively pump chemotherapy drugs out of cancer cells^3, 4^. Alterations in drug metabolism^5^ and DNA repair pathways^6^ can also contribute to chemoresistance. Multidrug resistance, on the other hand, involves the simultaneous resistance to multiple chemotherapy drugs, often due to the overexpression of various drug efflux pumps and changes in cellular signaling pathways. Understanding the underlying mechanisms of chemoresistance is paramount in developing effective strategies to overcome this challenge.

### The Role of Influx and Efflux Transporters in Chemoresistance of Breast Cancer

In breast cancer treatment, the challenge of influx/efflux transporter-induced chemoresistance is significant. Transporters like P-glycoprotein and multidrug resistance-associated proteins reduce the intracellular accumulation of chemotherapeutic agents, decreasing their effectiveness. For instance, Tamoxifen resistance in estrogen receptor-positive breast cancer is a major issue due to overexpressed drug efflux transporters such as P-glycoprotein, MRP, and breast cancer-resistant protein BCRP^7^. Research has shown that MCF7 cells exposed to tamoxifen developed reduced sensitivity and higher expression of drug efflux transporters^8^. Grasping how these transporters interact with chemotherapy drugs is vital for creating strategies to bypass resistance and enhance patient results.

#### ABCB1 (P-glycoprotein) is involved in the development of drug resistance in breast cancer:

ABCB1, also known as P-glycoprotein, is a protein encoded by the ABCB1 gene in humans. This protein is a member of the ATP-binding cassette (ABC) transporter superfamily, essential for transporting various molecules across cell membranes. Treatment of breast cancer often faces a significant obstacle in the form of resistance to drugs, such as doxorubicin (ADR), due to overexpression of P-glycoprotein (P-gp/ABCB1)^9^. RNA sequencing analysis has revealed the downregulation of GAS5 and upregulation of ABCB1 in ADR-resistant breast cancer tissues and cells^10^. Studies have indicated that overexpression of GAS5 enhances ADR sensitivity, induces cell apoptosis, and inhibits the function of ABCB1, offering the potential to overcome chemotherapy resistance in breast cancer patients^11^. The significance of P-glycoprotein in pharmacology and clinical medicine makes it a significant research and drug development target to overcome drug resistance in breast cancer.

#### ABC subfamily is involved in the development of drug resistance in breast cancer:

The ABCC is the subfamily of ABC. It is a protein group from the ATP-binding cassette (ABC) transporter superfamily^12–14^. These proteins are also involved in the transport of various molecules especially small chemical drugs across cell membranes, utilizing the energy from ATP hydrolysis to change protein conformation as a force in drug transport. The ABCC subfamily is known for its diverse substrate specificity, including the transport of ions, lipids, and chemical drugs. Members of this subfamily have been implicated in various physiological processes and diseases, making them an essential focus of molecular biology and medicine including the research in breast cancer drug resistance. An ABC transporter belonging to the ABCC subfamily has been identified as being localized at the plasma membrane, and it has been found to confer resistance to glyphosate^12–14^. This discovery sheds light on the molecular mechanisms underlying glyphosate resistance. It highlights the potential role of ABC transporters in this process for consideration in the research on breast cancer drug resistance.

#### ABCG2 is involved in the development of drug resistance in breast cancer:

ABCG2 is a key protein that maintains homeostasis and protects the body by transporting various molecules across cell membranes^15^. The role of ATP-binding cassette (ABC) transporters is of utmost importance in drug resistance and tissue protection from xenobiotics. These transporters are prominent in vital organs such as the intestine, liver, kidney, and placenta, which are critical in regulating the influx and efflux of various substances. A study found 75 potent inhibitors of the breast cancer resistance protein (BCRP) among FDA-approved drugs; thirteen had IC_50_ values ranging from 1.1 to 11 mM, with vemurafenib, dabigatran etexilate, and everolimus among the strongest^16^. Ongoing ABCG2 research continues to reveal its significant physiological and pharmacological roles in breast cancer drug resistance and treatment.

### The Application of the Series Circuit Mechanisms in Christmas-decorating-lighting for Developing Effective Therapeutics to Combat Breast Cancer Chemoresistance

#### The phenomenon in the Christmas-decorating-lighting system.

**Fig. 1.**
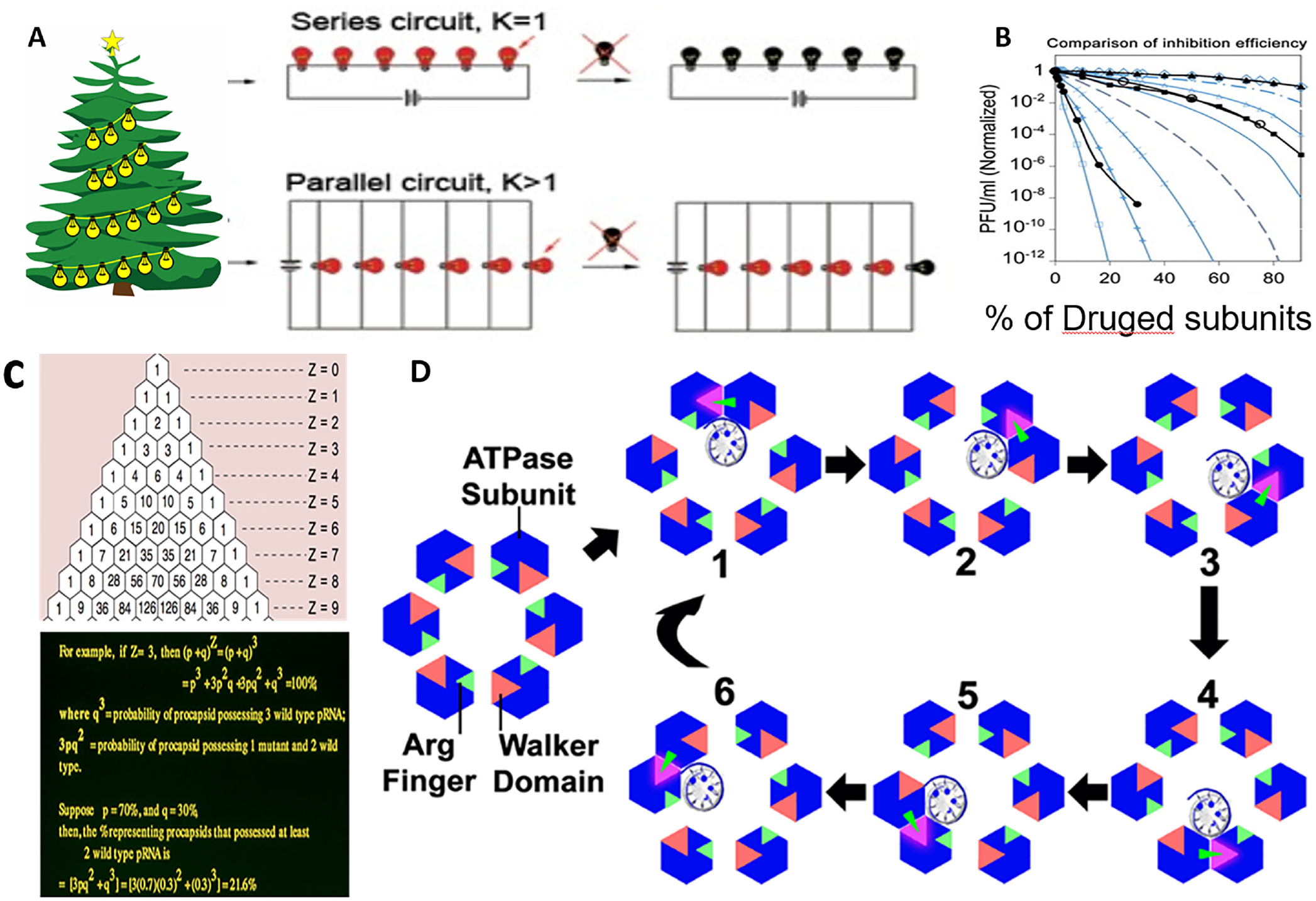
Mechanism and background for the development of potent drugs to target ABC and breast cancer cell receptors by applying the mechanisms of series circuit of Christmas bulge by targeting multiple homosubunits of the biological machine. A. The series circuit leads to one breaking of one bulge that will stop the entire lighting chain. B. Experimental data prove the concept stated in 1, dominating that the inhibition efficiency is a digital response to the stoichiometry of the homosubunit, agreed with the computation by Yanghui Triangle and binomial distribution^17, 23, 172^. And the parallel circuit. D. Many ATPase motors and regulators use the asymmetrical hexamer with a sequential action revolving mechanism that leads to the blocking of one subunit, which will stop the entire machine with revolving mechanics. In sum, the effectiveness of drug inhibition is intrinsically linked to the stoichiometry of the target biological complex^173^. In this framework, the parameter K in A, representing the drugged subunit, plays a pivotal role in determining drug potency. When the subunits targeted by the drug operate sequentially with K=1, the system resembles a series circuit of Christmas lights; a single faulty bulb can disrupt the entire chain. Conversely, when K>1, it is similar to a parallel circuit as in the traditional drug design with one drug target to one independent location, where the overall system will only be wholly disrupted if all bulbs in the parallel circuit malfunction.

Christmas tree lights can be wired with two kinds of circuits: parallel circuits and series circuits (Fig. 1). If one bulb burns out in a parallel circuit, the other bulbs will continue to function in lighting because electricity can flow along multiple paths to the ground. If one bulb burns out in a series circuit, the entire circuit will turn off. In traditional drug development, one chemical drug is explicitly designed to bind one target. This traditional approach is similar to the parallel circuit. Each chemical molecule will bind to one substrate independently; one substrate molecule is inactive, and the other is still active since they are independent. We have proposed and tested innovative therapeutic strategies for the series circuit mechanism. In this case, if one substrate is targeted by one drug, the system will turn off. Thus, such a drug will be very efficient. To mimic the series circuit, the substrate as a target should contain multi-homomeric units that mimic the series circuit with many bulges. In addition, all the subunits should work sequentially; that is, all the subunits should work coordinately or cooperate, not independently. The biological complex is supposed to contain Z units of the homomer complex. The additional requirement is that all the subunits work sequentially and not independently thus K = 1, where K is the number of drugged subunits required to block the function of the machine, a condition that is like a series of the electrical circuit of the Christmas decoration bulb: the breaking of one single lighting bulb causes the entire lighting system to lose power. This is because the coordination of all the homologous subunits will lead to the consequence that if one subunit is inactivated, the entire system will turn off. It has been reported that in many ATPases, such as the viral DNA packaging motor, the bacterial genome transporter, and the Holliday junction, these machines show six (hexamer) subunits that work sequentially. Many of the ABC drug efflux pump are homo-polymers, and a sequential action mechanism is utilized. Thus, K equals 1, and since Z > 1, the inhibition effect follows a power law of Z, leading to an amplified potency. Due to this potency, the programmability and multivalent nature of RNA nanoparticles facilitate the incorporation of targeting ligands, and co-deliver of siRNA, miRNA, or drugs, which can silence the drug effect pump with high efficiency (Fig. 1)^17, 18^.

### Mimic the Christmas lighting series circuit mechanism to develop potent anti-breast cancer drugs targeting the homo-multimer ABC

Chemotherapeutic failure is often due to chemoresistance, involving factors like ABC transporter overexpression, drug-target interaction modifications, and altered DNA repair. The breast contains some ABC drug efflux pumps that are homo-polymers with a sequential action mechanism. Thus, K=1 and Z > 1, leading to the possibility of developing potent drugs for overcoming breast cancer resistance. Due to this potency, the programmability and multivalent nature of RNA nanoparticles facilitate the incorporation of targeting ligands, and co-deliver of siRNA, miRNA, or drugs, which can silence the drug efflux pump with high efficiency. The inhibition efficacy in drug resistance breast cancer will follow a power law (Fig. 1).

To address that question, ongoing strategies include inhibiting ABC transporters, utilizing chemosensitizers, and employing nanotechnology for targeted delivery. Promisingly, siBmi-1 interventions target the Bmi-1 oncogene linked to HCC malignancy and Cisplatin resistance, potentially curbing cell proliferation and silencing resistance genes, thereby improving chemotherapy efficacy^19^. Chemoresistance poses a significant hurdle in cancer treatment, mainly due to the overexpression of ATP-binding cassette (ABC) transporters that expel chemotherapeutic agents from cancer cells, diminishing their effectiveness. Extensive research has aimed at creating inhibitors for these transporters to increase drug retention and efficacy. However, the clinical use of such inhibitors is often constrained by their non-specificity and related toxicity, limiting their therapeutic benefit. Therefore, the development of more selective and safer inhibitors is a key focus in the fight against chemoresistance^20, 21^. A novel method for creating effective inhibitory drugs targets biological machines made of multi-homologous subunits working sequentially. Inspired by this, researchers are developing antiviral drugs to inhibit viral DNA-packaging motors. This strategy disrupts multisubunit systems with sequential actions, like disabling one bulb in a Christmas lights series, causing the entire chain to shut down. The study conducted by Mark Trottier and Peixuan Guo^22^ demonstrated that the incorporation of mutant subunits into the multi-homologous viral DNA packaging motor resulted in complete inhibition of viral assembly. The observed inhibition substantially reduced plaque-forming units, effectively decreasing the count by 7–8 orders of magnitude. This finding highlights the potential of targeting viral DNA packaging mechanisms as a strategy for antiviral therapy^12–14^.

As previously discussed, the high potency of these drugs is due to a mechanism that follows a power law based on the number of homo-multimers, enhancing their effectiveness. RNA nanoparticles’ programmable and multivalent properties allow for the combined delivery of targeting ligands, RNA interference (RNAi), and chemical drugs within a single nanoparticle. RNA nanotechnology offers a promising approach for displaying breast cancer ligands on extracellular vesicles, enabling the delivery of chemical drugs and RNAi to combat chemoresistance in breast cancer. This innovative technique draws inspiration from the successful use of the P-gp for liver cancer^13, 14^ and the silencing of drug efflux pumps ABC by miR122 to silence the P-gp efflux pump^13, 14^. By leveraging RNA nanotechnology, researchers aim to enhance the efficacy of breast cancer treatment by overcoming resistance mechanisms and improving targeted delivery of therapeutic agents. Recent research indicates that ABC drug efflux pumps, possibly all, are multi-homologous subunits functioning sequentially via alternative ATP binding. Furthermore, the asialoglycoprotein receptor (ASGPR), a key biomarker for hepatocellular carcinoma (HCC), has been successfully targeted with ligand-conjugated siRNA. This method has shown precise gene inhibition after subcutaneous administration. Additional studies suggest siRNA-based therapies could overcome chemoresistance, offering promising avenues to improve cancer treatment efficacy^23^.

### Mimic the Christmas lighting series circuit mechanism to develop potent anti-breast cancer drugs targeting the homo-multimer nicotinic acetylcholine receptor

According to this theory, many cellular molecules can be utilized in future treatments. One example of a target in cancer cells that meets these criteria and is suitable for clinical treatment is the nicotinic acetylcholine receptor (nAChRs). nAChRs are specialized proteins that act as ligand-gated ion channels^24^. There are two primary categories of receptors: muscle receptors located at skeletal neuromuscular junctions, which aid in neuromuscular communication, and neuronal receptors dispersed throughout the peripheral and central nervous systems, essential for quick synaptic transmission. nAChRs have a pentameric structure, indicating they consist of five subunits^25^. The human proteome contains 16 distinct subunits of nicotinic acetylcholine receptors. Of these, 11 are found in neurons (α2–7, α9, α10, β2–4) and 5 are found in muscles (α1, β1, γ, δ, and ε). The human genes are *CHRNA1–7, CHRNA9, CHRNA10, CHRNB1–4, CHRNG, CHRND*, and *CHRNE*.

**Fig. 2.**
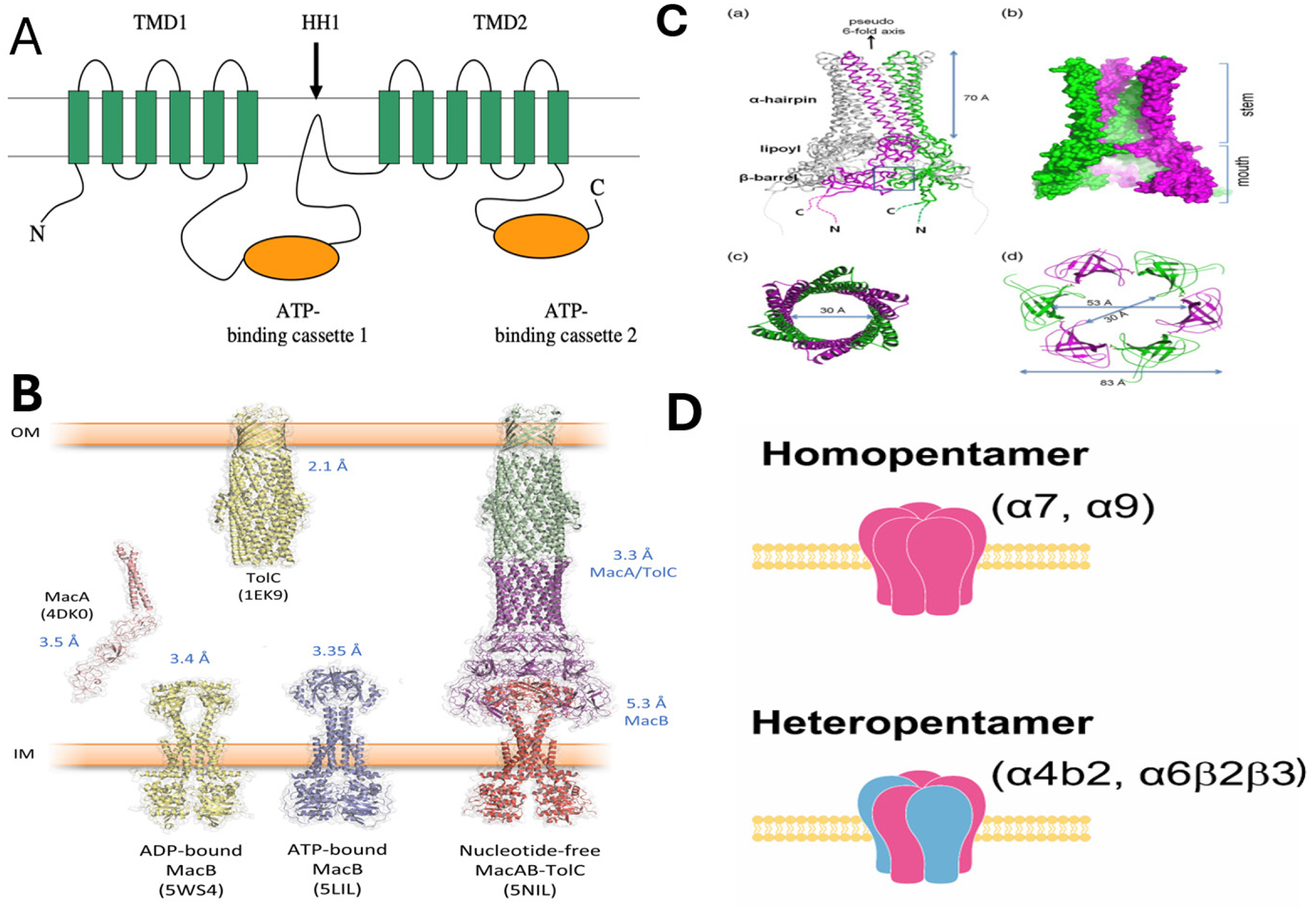
The typical structure and stoichiometry of ABC drug transporters and breast cancer cell receptors showing the formation of the pore with multiple homologous subunits that can be used as potent drug targets. A. The protein structures of ABC A-subfamily. The two halves of the protein, each containing two transmembrane domain TMD αand an ATP binding cassette, are separated by a characteristic hydrophobic loop HH1 (arrow) that dips into the membrane^174^. C. The structure of the breast cancer cell nicotine receptor α^33, 175, 176^.

As allosteric proteins, nAChRs can change one receptor part when a ligand binds to another site. The receptor’s extracellular domain holds binding sites for various ligands, and these bindings can alter the receptor’s function through allosteric means. nAChRs are essential in regulating resting membrane potential, influencing synaptic transmission modulation, and supporting fast excitatory transmission. Among them, the α7 and α9 nicotinic acetylcholine receptors are homopentameric ion channels within the Cys-loop superfamily, known for their low likelihood of opening, high permeability to calcium, and swift desensitization^26, 27^. The α7 subunit is the most abundantly expressed and distributed in the human brain. In contrast, the neuronal heteropentamers are composed of two α units (ranging from α2 to α6) and three β units (ranging from β2 to β4). These heteropentameric receptors feature two acetylcholine-binding sites, each between an α and a β subunit. In contrast, homopentameric receptors possess five binding sites located at the interface between two subunits^28–30^. Recent studies have shown that the α7 and α9 nicotinic acetylcholine receptors are implicated in developing various human cancers^31–36^. These receptors are critical in cell signaling and communication processes and are key elements that help cells respond to their environment and coordinate with each other (Fig. 2). For example, the α9 nAChR can interact with the HER2 receptor and influence breast cancer treatment^37^. These homomeric molecular structures are involved in cancer formation, highlighting the potential for targeted therapy. Our previous research papers also demonstrate that inhibiting the activity of a single subunit of the α9 nAChR can effectively suppress tumor growth^38, 39^. It is currently well-established that many natural compounds have been confirmed to inhibit the expression of nicotinic acetylcholine receptors (Table 2). Understanding how these receptors contribute to cancer can pave the way for new treatment strategies and improve patient outcomes.

The requirement of homologous subunit in the drug-resistant machine or other ATP-binding biomotors does not assume that the monomers do not change shape in any way upon drug binding. As a general mechanism of complex assemblies, allostery plays a major role in the biological function. Here, the analogies are the application that in the homo-subunit, there is always an included chance and probability to hit the substrate as long as the drug develops to be one of the subunits, no matter if it is the original structure or the allostery form.

### Current understanding of mechanisms inducing breast cancer drug resistance

**Fig. 3.**
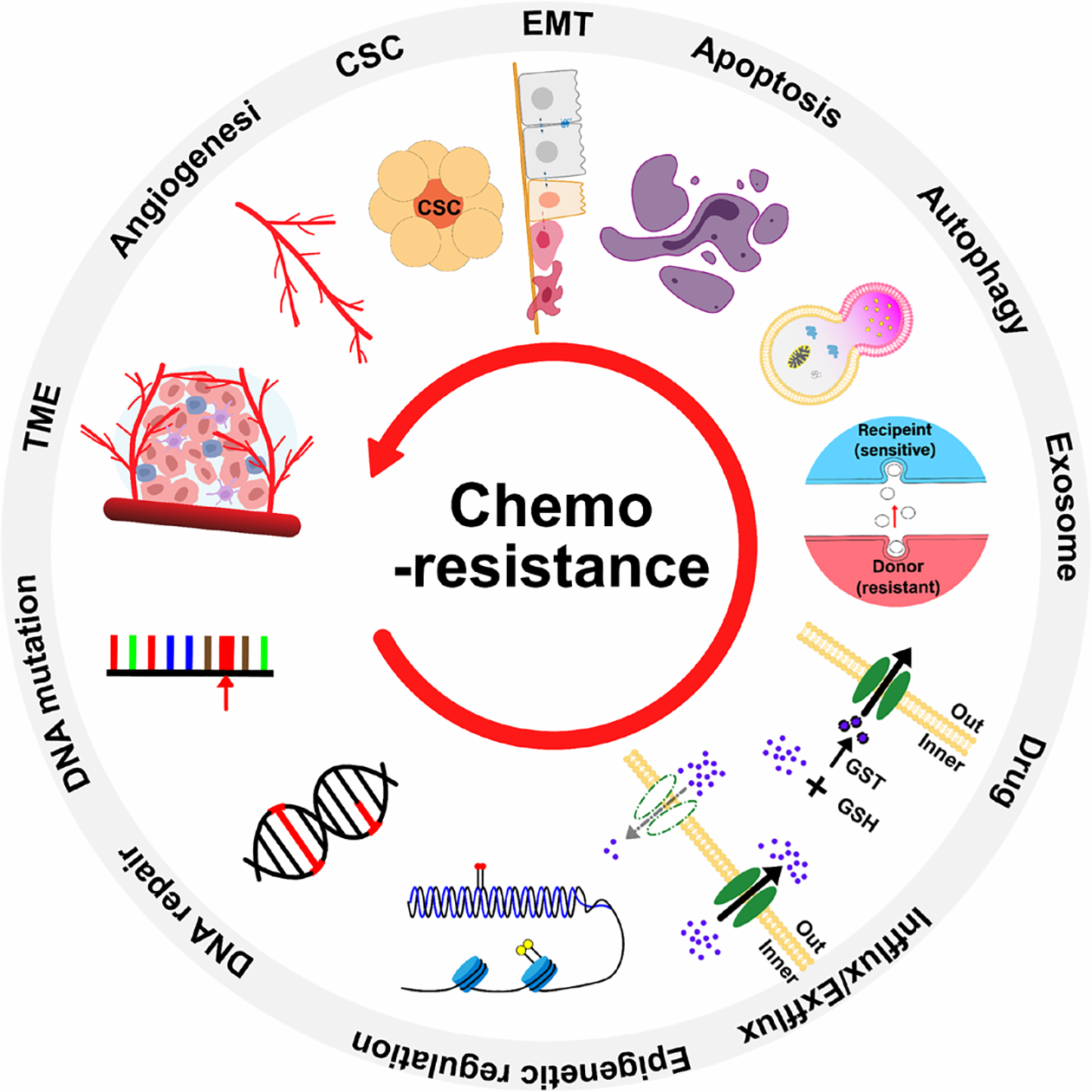
Current Understanding of Mechanisms Inducing Chemoresistance. The development of chemotherapy resistance in cancer cells progresses from alterations in cellular genes (including DNA repair and epigenetic regulation) to changes in intracellular proteins involved in drug absorption and metabolism (such as ABC transporters), leading to modified drug tolerance (e.g., induction of apoptosis and autophagy), influencing cancer cell characteristics (such as EMT and CSC), and ultimately altering the tumor microenvironment and angiogenesis.

Recent discoveries have shed light on the mechanisms underlying chemoresistance in breast cancer treatment. It has been found that variations in drug processing, activation of survival pathways, and modifications in the tumor microenvironment play crucial roles in conferring resistance to chemotherapy. These changes encompass modifications in drug-processing enzymes and carriers, activation of pathways such as PI3K/AKT/mTOR, and modifications in hypoxia and extracellular matrix within the tumor environment. Grasping these mechanisms is vital for combating chemoresistance and enhancing breast cancer treatment (Fig. 3).

#### Epigenetic regulation is involved in breast cancer drug resistance.

A novel finding in breast cancer research is discovering epigenetic regulation of treatment resistance^40^. This breakthrough has significant implications for developing more effective treatment strategies for breast cancer patients. Epigenetic dysregulation is associated with cancer^41, 42^, involving patterns like DNA methylation, histone modifications, miRNA, abnormal lncRNA expression, and small nucleolar RNA. Specifically, lncRNA influences breast cancer, exhibiting oncogenic and tumor-suppressive potential and contributing to resistance to various treatments. It also serves as a non-invasive diagnostic biomarker and is relevant in cancer stem cells of breast tumors^43^.

#### The involvement of the hypoxia-inducible pathway in breast cancer drug resistance has been well-documented.

The hypoxia-inducible pathway plays a significant role in the development of chemoresistance in breast cancer. Hypoxia, or low oxygen levels, within the tumor microenvironment, activates this pathway, leading to the upregulation of various genes that promote cell survival and resistance to chemotherapy. These genes include those encoding drug efflux pumps^44^, anti-apoptotic proteins^45^, and DNA repair enzymes. As a result, breast cancer cells become less responsive to chemotherapy^46, 47^, making treatment less effective. Understanding the mechanisms of hypoxia-induced pathways in chemoresistance is imperative for the development of effective targeted therapies to address this significant challenge in breast cancer treatment.

#### Autophagy-induced chemoresistance has been observed in breast cancer.

Recent research indicates that malonate plays a dual role in cancer metabolism^48, 49^. It has been found to induce the production of reactive oxygen species, leading to cell apoptosis and protection from ischemia-reperfusion injury. High doses of malonate induce apoptosis, while low doses promote autophagy and increase resistance to chemotherapy drugs^48^. Additionally, malonate increases the stability of the p53 protein and promotes autophagy, highlighting its cancer-promoting role in p53-induced autophagy^48^.

### Exploring the advantages of RNA nanotechnology in anti-breast cancer drug development

RNA nanotechnology offers programmable and multifunctional design capabilities for complex nanostructures, enabling precise control over shape and function^50^. Its biocompatibility and biodegradability make it an attractive material for biomedical applications, including innovative therapies such as RNA-based vaccines and gene therapy. The ability to design RNA molecules with diverse functions expands the potential of RNA nanotechnology across biomedicine, biotechnology, and nanotechnology.

#### RNA nanomaterials’ biocompatibility, biodegrad-ability, and stability ensure their safety and efficacy in treating breast cancer.

RNA nanotechnology has shown promise in diverse medical and nanomedicine applications. When developing RNA-based nanomaterials, it’s vital to consider biocompatibility, biodegrad-ability, and stability to ensure their safety and efficacy in biomedical applications. xtracellular vesicles (EVs) hold potential as therapeutic delivery vehicles, but challenges such as cost and target specificity persist. Plant-derived EV-like nanovesicles offer biocompatibility through various administrations. Using RNA nanogels on ginger-derived EV-like nanovesicles (GDENs) with folate as the ligand, we successfully delivered survivin siRNA to tumor models, leading to tumor growth inhibition^51^. This highlights the potential of GDENs as an economical siRNA delivery system.

#### Structural versatility and programmability of RNA for breast cancer drug development.

RNA nanotechnology’s versatile structure and programmability make it a powerful tool in nanomedicine and nanobiotechnology. It allows for the design of diverse nanostructures with specific functions, self-assembling into complex architectures, and incorporating functional elements for specific biological activities. RNA nanotechnology enables dynamic control over nanostructure assembly/disassembly and molecular interactions within biological systems, offering the potential for targeted drug delivery, imaging, and sensing applications in breast cancer treatment^52^.

#### Target delivery and cellular uptake of RNA nanomedicines and their anti-breast cancer effects.

**Fig. 4.**
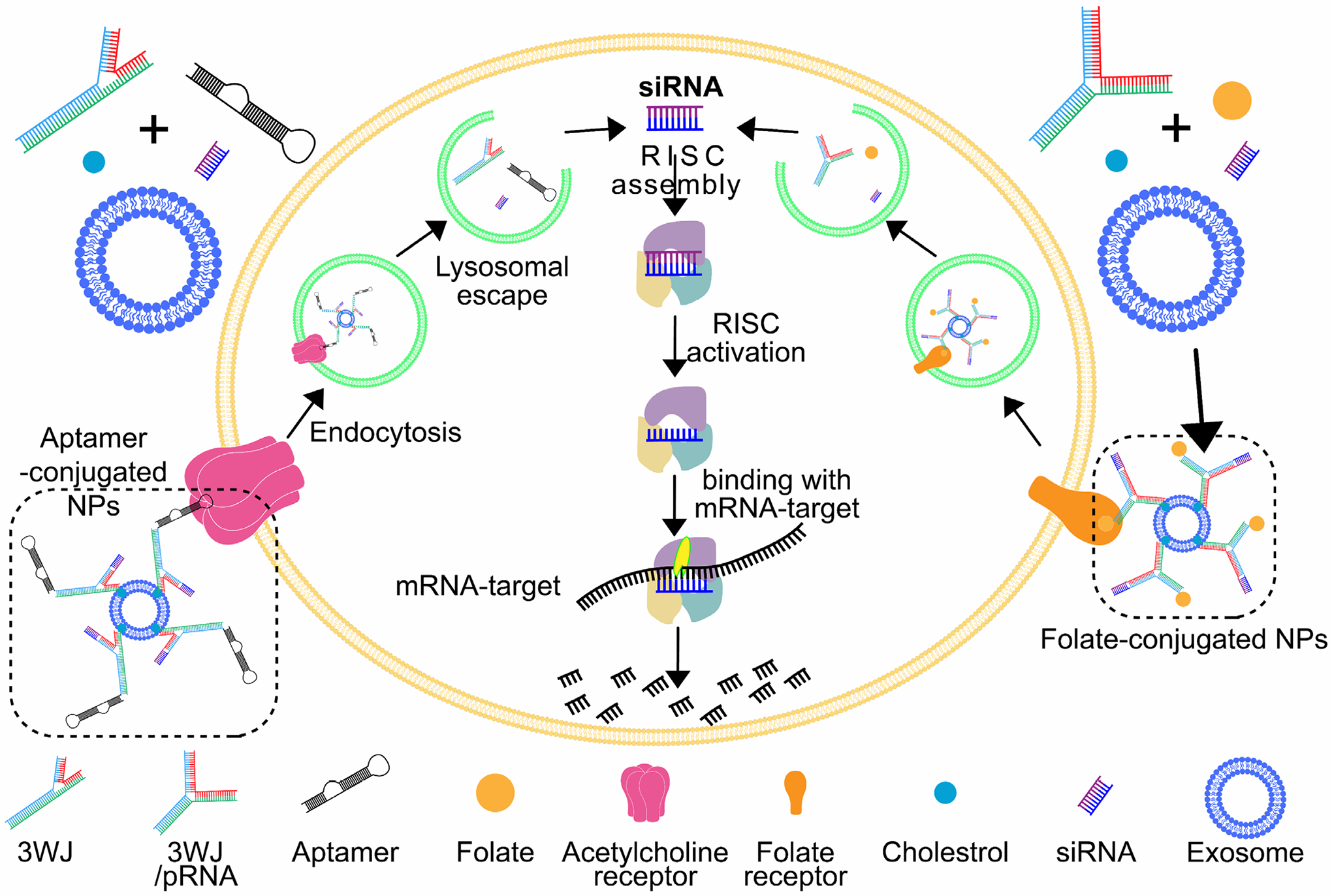
RNA nanotechnology-mediated gene therapy for liver cancer. The antigen specificity of aptamers can be utilized to target RNA nanomedicines specifically to tumor cells. These RNA nanoparticles can bind to extracellular vesicles through lipophilic molecules such as cholesterol. Through the endocytosis process between cancer cells and extracellular vesicles, RNA nanomedicines can be delivered into cancer cells, where the siRNA carried by the RNA nanomedicines can enter the cells and exert its therapeutic effects. Small interfering RNA (siRNA), typically consisting of 20–25 base pairs with two-nucleotide overhangs at the 3’ end, are specialized RNA molecules that play a crucial role in gene silencing through the RNA interference (RNAi) mechanism. This process involves the recognition of specific sequences by the Dicer/RISC complex, leading to the targeted cleavage of messenger RNA (mRNA) via site-specific complementary base pairing. The ability of siRNAs to selectively silence gene expression holds significant potential for therapeutic applications and advancements in molecular biology research.

One critical advantage of RNA nanotechnology is its potential for targeted delivery of therapeutic agents to specific cells or tissues. Researchers can achieve precise targeting of therapeutic payloads by engineering RNA nanoparticles to display ligands or aptamers that can bind to cell surface receptors or other molecular targets^53, 54^. This targeted delivery approach not only enhances the efficacy of therapeutic agents but also minimizes off-target effects, thereby improving the safety profile of the treatment (Fig. 4).

Moreover, RNA nanoparticles can be designed to encapsulate and protect fragile cargo, such as small interfering RNA (siRNA) or microRNA, from degradation in the extracellular environment^53^. This protective function is precious for nucleic acid-based therapeutics, which are susceptible to enzymatic degradation and rapid clearance from the circulation. In addition to targeted delivery, RNA nanotechnology also offers opportunities to enhance the cellular uptake of therapeutic agents. Through rational design and engineering, RNA nanoparticles can be tailored to interact with cellular uptake pathways and facilitate internalization into TNBC cells^38^. This capability is significant for delivering nucleic acid-based therapeutics, which typically require efficient uptake into the cytoplasm of target cells to exert their therapeutic effects. Furthermore, RNA nanoparticles can be modified with cell-penetrating peptides^55^ or other functional moieties to enhance their cellular uptake and endosomal escape^56^. The adjustments mentioned above have the potential to greatly enhance the internal transport of therapeutic RNA molecules, effectively addressing challenges related to cellular absorption and movement within the cell.

#### Reduced immunogenicity and toxicity.

Recent advancements in RNA nanotechnology have focused on addressing concerns about immunogenicity and toxicity. Designing RNA nanoparticles that mimic natural structures found in the body, such as extracellular vesicles^57^ or virus-like particles^58^, can reduce immunogenicity, making them more suitable for therapeutic use. Modifications to the RNA sequence and structure can also help mitigate immune recognition. Efforts to optimize RNA nanoparticle chemical composition and delivery methods have minimized potential toxic effects. Additionally, using biocompatible materials for RNA encapsulation and delivery vehicles has contributed to the overall safety profile of RNA nanotechnology^59^.

Overall, the ongoing efforts to reduce the immunogenicity and toxicity of RNA nanotechnology have paved the way for its broader application in biomedicine. With continued research and development, RNA nanomaterials hold great potential for targeted drug delivery, gene therapy, and diagnostic imaging with improved safety and efficacy.

### Enhancing Biodistribution and Pharmacokinetics in Antitumor Drug Development Strategies

**Fig. 5.**
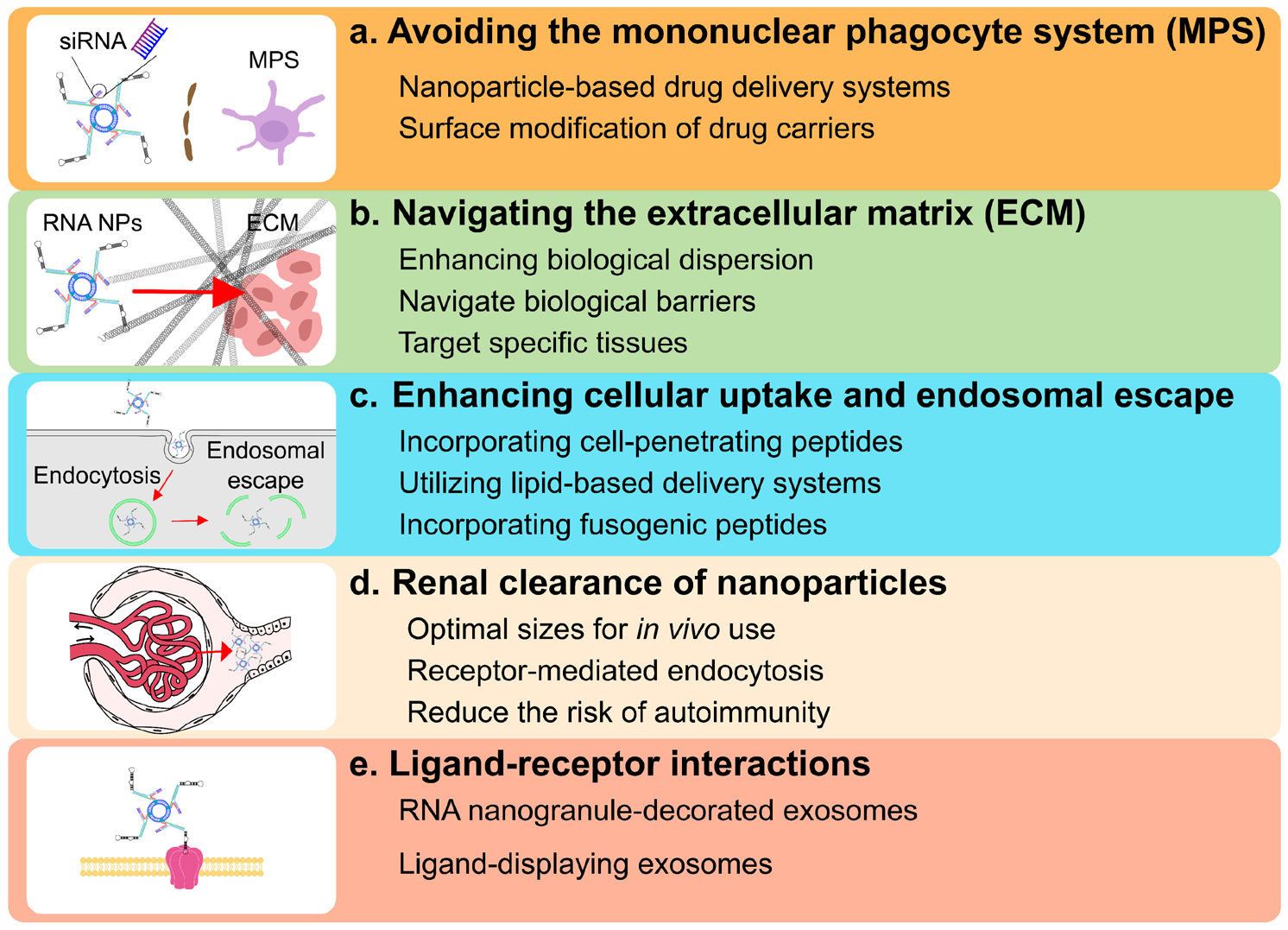
Enhancing Biodistribution and Pharmacokinetics in Antitumor Drug Development Strategies. To enhance the strategies for developing anti-tumor drugs in terms of biodistribution and pharmacokinetics, several key approaches must be considered, including: A. Avoiding the mononuclear phagocyte system (MPS) by improving nanoparticle drug delivery systems and surface modifications of drug carriers to minimize MPS uptake and enhance drug accumulation in tumor tissues; B. Precision navigation of the extracellular matrix (ECM) through the adaptive use of RNA molecules, enabling scientists to create advanced nanocarriers that traverse biological barriers and accurately target specific tissues; C. Enhancing cellular uptake and endosomal escape by optimizing the design of RNA nanoparticles, such as incorporating cell-penetrating peptides or utilizing lipid-based delivery systems; and D. Optimizing the renal clearance rate of nanoparticles, ensuring that RNA nanoparticles have an optimal size for in vivo use—greater than 10 nm for rapid renal excretion yet small enough to enter cells via receptor-mediated endocytosis, thereby evading macrophage capture and reducing the risk of autoimmune reactions by minimizing interactions with negatively charged membranes.

RNA nanotechnology holds great promise for overcoming breast cancer chemoresistance, but its clinical application is hindered by suboptimal biodistribution and pharmacokinetics. Several strategies can be employed to address this challenge and improve the delivery of RNA nanotechnology to tumor sites (Fig. 5).

#### Avoiding the mononuclear phagocyte system (MPS).

Chemoresistance remains a significant challenge in cancer treatment, and one potential contributing factor is the mononuclear phagocyte system (MPS). The MPS, which includes monocytes, macrophages, and dendritic cells, plays a critical role in the clearance of foreign substances, including chemotherapeutic agents^60^. This can lead to reduced drug concentrations at the tumor site and subsequent treatment failure. Therefore, strategies to avoid the MPS and enhance drug delivery to the tumor are of great interest. Various approaches, such as nanoparticle-based drug delivery systems^61^ and surface modification of drug carriers, are being explored to minimize MPS uptake and improve drug accumulation in tumor tissues^62^. The FDA has approved five siRNA-based therapeutic drugs: patisiran, givosiran, lumasiran, inclisiran, and vutrisiran. Delivering siRNA faces challenges such as membrane impermeability and enzymatic degradation. Nanocarriers, especially lipid nanoparticles, promise to improve siRNA delivery with minimal toxicity^63^.

#### Navigating the extracellular matrix (ECM).

Studying RNA nanotechnology in the extracellular matrix (ECM) has significant potential for enhancing biological dispersion^64^. This technology can revolutionize drug delivery and tissue engineering using RNA-based nanomaterials to improve the body’s dispersion and retention of therapeutic substances. By harnessing the adaptable nature of RNA molecules, scientists can create advanced nanocarriers to navigate biological barriers and target specific tissues accurately, thereby increasing the effectiveness and safety of treatments for various diseases and disorders^65^. The progression of RNA nanotechnology offers compelling potential for enhancing biological dispersion through ECM manipulation.

#### Enhancing cellular uptake and endosomal escape.

It is imperative to improve cellular uptake and endosomal escape, thereby enhancing biodistribution by optimizing the design of RNA nanoparticles, such as incorporating cell-penetrating peptides^55^ or utilizing lipid-based delivery systems^66^. Lipid-based nanocarriers like liposomes, lipid nanoparticles, and lipid nanoemulsions can encapsulate and deliver RNA-based therapeutics. However, researchers encounter challenges related to the toxicity of cationic lipids and PEGylated lipids. It is imperative to comprehend these issues as they play a critical role in the progression of RNAi therapy towards clinical application. Additionally, strategies to promote endosomal escape, such as incorporating fusogenic peptides^67, 68^, can further improve the intracellular delivery of RNA nanotechnology. These advancements will ultimately contribute to developing more effective RNA-based therapeutics with improved biodistribution and cellular uptake.

#### Renal clearance of nanoparticles.

Renal clearance of RNA nanoparticles offers a promising strategy for reducing systemic toxicity^69, 70^. These nanoparticles can be efficiently cleared from the bloodstream, minimizing off-target effects and accumulation in non-target tissues. With optimal sizes for in vivo use, they can evade capture by macrophages and reduce the risk of autoimmunity. Depending on nucleotide modification, their in vivo half-life can extend to 5–12 hours^34^, compared to 0.25–0.75 hours for unmodified siRNA^58^. When systemically injected into mice with cancer, pRNA-3WJ nanoparticles selectively target cancer cells with minimal build-up in healthy organs, indicating potential as a promising cancer treatment option with reduced impact on healthy tissues^34–36,59–63^. Many RNA nanocarriers are ratchet-shaped to move quickly to tumors^35,36,59^ and have rubber-like elastic properties^64^, enabling them to navigate through cancer vasculature under pressure, enhancing the enhanced permeability and retention (EPR) effect.

#### Ligand-receptor interactions.

RNA-modified extracellular vesicles enhance target specificity and protect loaded contents from degradation and non-specific interactions^57, 71^. RNA nanogranule-decorated extracellular vesicles facilitate targeted delivery and rapid renal clearance while minimizing toxicity. Displaying RNA ligands on extracellular vesicles enables tracking of treatment outcomes and offers potential for cancer therapy^72^. RNA nanoparticles with ligands prevent off-target binding and target specific cancer cells through ligand-receptor interactions. Folic acid-displaying extracellular vesicles effectively deliver siRNA to cancer cells, leading to tumor regression and redefining folic acid’s potential as a cancer-targeting ligand for human cancer therapy^73^.

### The Utilization of RNA-Mediated Gene Therapy for the Treatment of Breast Cancer

**Fig. 6.**
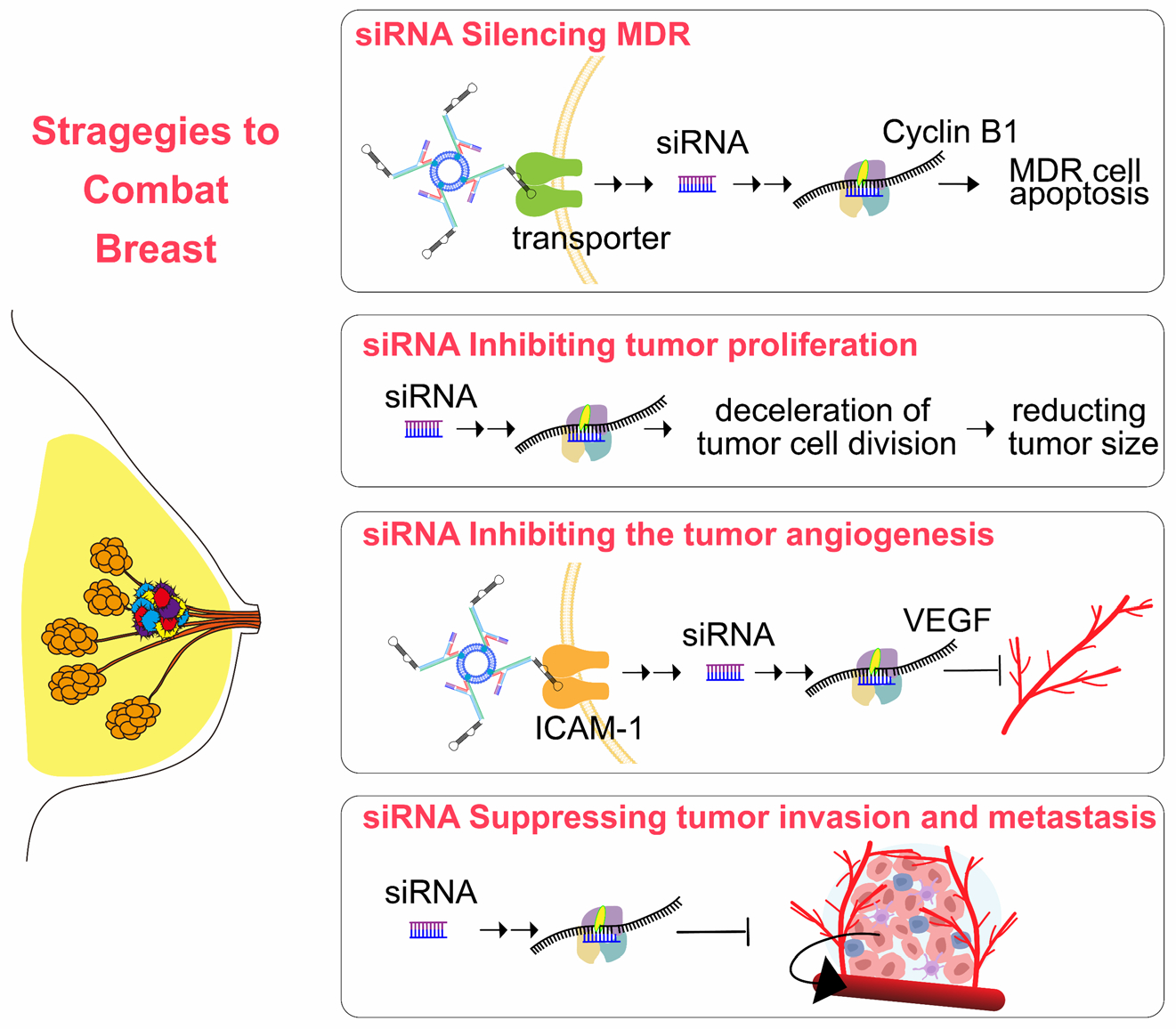
RNA-Mediated Gene Therapy for Breast Cancer Treatment: A Promising Approach. RNA-mediated gene therapy represents a promising strategy for the treatment of breast cancer. It aims to inhibit tumor growth and enhance patient outcomes by targeting specific genes involved in cancer progression. By delivering RNA-based therapeutics, such as small interfering RNA (siRNA) or microRNA, this approach can achieve therapeutic goals by silencing multidrug resistance (MDR) genes, inhibiting tumor proliferation, and preventing tumor angiogenesis.

RNA-mediated gene therapy is a promising approach for the treatment of breast cancer^74^. This therapy can inhibit tumor growth and improve patient outcomes by using RNA molecules to target specific genes involved in cancer progression. Through the delivery of RNA-based therapeutics, such as small interfering RNA (siRNA) or microRNA, the expression of oncogenes can be suppressed, leading to a reduction in cancer cell proliferation and metastasis. RNA-mediated gene therapy can also enhance cancer cells’ sensitivity to conventional treatments, such as chemotherapy and radiation therapy (Fig. 6).

#### siRNA as an approved therapeutic: siRNA Silencing MDR.

SiRNA (small interfering RNA) silencing of multidrug resistance (MDR) genes has emerged as a promising strategy for enhancing the efficacy of breast cancer therapy^75^. MDR genes play a critical role in the development of resistance to chemotherapy drugs, leading to treatment failure and disease progression in breast cancer patients. In a recent study^76^, pH-sensitive carbonate apatite nanoparticles were designed and developed to efficiently deliver siRNA targeting ABCG2 and ABCB1 gene transcripts across the cell membrane. This innovative approach enabled the knockdown of the cyclin B1 gene, leading to the induction of apoptosis in synergy with anticancer drugs. This approach holds great potential for improving the outcomes of breast cancer treatment and addressing the challenges posed by drug resistance.

#### siRNA as an approved therapeutic: siRNA Inhibiting tumor proliferation.

Small interfering RNA (siRNA) has emerged as a promising therapeutic strategy for inhibiting tumor proliferation in breast cancer. By specifically targeting genes that play a critical role in tumor growth and progression, siRNA demonstrates the ability to inhibit their expression efficiently. This targeted gene silencing results in a deceleration of tumor cell division and a subsequent reduction in tumor size^69, 77, 78^. This approach holds great potential for the development of more targeted and personalized breast cancer therapies, intending to improve patient outcomes and reduce the burden of this devastating disease^79^.

#### siRNA as an approved therapeutic: siRNA Inhibiting the tumor angiogenesis.

Recent advancements in breast cancer therapy have focused on targeting tumor angiogenesis, the process of blood vessel formation that supports tumor growth and metastasis. One promising approach is using siRNA to inhibit the expression of key genes involved in angiogenesis^80, 81^. This innovative strategy holds great potential for disrupting the tumor’s blood supply and ultimately impeding its growth. The previous study revealed that Lipocalin 2 (Lcn2) shows potential as a therapeutic target and diagnostic biomarker for breast cancer ^(80)^. This study introduces a novel lipid nanoparticle (LNP) small interfering RNA (siRNA) delivery system targeting intercellular adhesion molecule-1 (ICAM-1) for triple-negative breast cancer (TNBC). This ICAM-1-targeted Lcn2 siRNA encapsulated LNP (ICAM-Lcn2-LP) exhibits higher binding to TNBC cells than non-tumorigenic cells. It effectively reduces Lcn2 levels and vascular endothelial growth factor (VEGF) production, reducing angiogenesis in vitro and in vivo, essential for tumor progression. The targeted anti-angiogenic therapy developed in this study may have clinical implications in inhibiting TNBC progression.

#### siRNA as an approved therapeutic: siRNA Suppressing tumor invasion and metastasis.

The siRNA has shown significant promise in suppressing tumor invasion and metastasis^82^. By specifically targeting and silencing genes involved in these processes, siRNA can effectively inhibit cancer cells’ pathways to spread and invade surrounding tissues. This targeted approach enhances the precision of cancer treatment and minimizes the adverse effects typically associated with conventional therapies. Recent studies have demonstrated the potential of siRNA in reducing metastatic activity in various cancer models^83^, underscoring its viability as a therapeutic strategy in oncology. Continued research and clinical trials are essential to fully understand the scope and efficacy of siRNA-based interventions in combating cancer metastasis.

### microRNA presents multivalent expression changes in and against cancer

Recent studies have revealed significant changes in miRNA expression levels in breast cancer^84^, underscoring its regulatory complexity and potential for therapeutic intervention. Research in Guo’s lab targets various cancers, including TNBC breast cancer^71, 85^, glioma^86^, and acute lymphoblastic leukemia^87^, aiming to enhance understanding and treatment. Advances in RNA nanotechnology show promise in inhibiting tumor invasion and metastasis, especially in breast cancer^88^. A recent RNA nanotechnology study delivered anti-miR-21 to inhibit TNBC growth in mice^71^, demonstrating resistance to degradation, structural integrity, and minimal healthy organ accumulation. This innovative approach has the potential to make a substantial impact on breast cancer outcomes and ultimately improve patient survival rates (Table 3).

#### microRNA Overcoming Gene-Induced Multidrug Resistance (MDR) in Chemotherapy.

The miRNA, or microRNA, has shown potential in circumventing chemoresistance by targeting genes involved in MDR^89, 90^. By modulating the expression of these genes, miRNA can help overcome the mechanisms that contribute to MDR, thereby enhancing the efficacy of chemotherapy. Prior research has demonstrated that miRNA-765 is elevated in MDR gastric cancer (GC) cell lines and chemotherapy-resistant patient samples. Downregulation of miRNA-765 enhances GC cell sensitivity to anticancer drugs, while upregulation has the opposite effect. MiRNA-765 also hinders drug-induced cell apoptosis and upregulates MDR-related gene expression, specifically targeting the tumor suppressor gene alkaline leucine zipper ATF-like transcription factor 2. These findings indicate that miRNA-765 may exacerbate MDR in GC cells by targeting ATF-like transcription factor 2^91^. This approach holds promise for addressing the challenge of chemoresistance in cancer treatment and may lead to developing novel therapeutic strategies.

#### microRNA Overcoming Tumor proliferation and apoptosis.

MicroRNA (miRNA) has been widely recognized for its significant role in regulating tumor proliferation and apoptosis in breast cancer. Studies have demonstrated that specific miRNAs (such as miRNA-138-5p) act as tumor suppressors by inhibiting the proliferation of cancer cells and promoting their programmed cell death or apoptosis^92^. In a separate study^93^, researchers sought to identify prospective drugs for treating triple-negative breast cancer (TNBC), specifically focusing on compound 4. This compound is a thiosemicarbazone derivative designed to target the MDA-MB 231 breast cancer cell line. Combining cisplatin and compound 4 showed promise in combating TNBC by enhancing p53 phosphorylation, inducing Bax, reducing Bcl2 levels, increasing PARP cleavage, and modulating miRNA expression profiles. Specifically, there was an increase in miR-125a-5p and miR-181a-5p expression^93^. New research suggests that miRNAs, particularly miRNA106-a, are promising therapeutic targets for battling breast cancer. This finding warrants further investigation into miRNAs as potential treatment options^94^, offering a promising avenue for developing novel anticancer strategies.

#### The Role of microRNA in Inhibiting Tumor Angiogenesis.

In the context of breast cancer therapy, the inhibition of tumor angiogenesis through miRNA manipulation has emerged as a promising area of research^95^. For example, phosphatase and tensin homolog (PTEN) are vital tumor suppressor proteins in cancer prevention. Its primary function involves inhibiting the oncogenic PI3K/Akt/mTOR signaling pathway, which regulates cell growth and promotes cell survival. PTEN dephosphorylates PIP3 to counteract this pathway, maintaining cellular balance. Mutations or loss of PTEN can result in uncontrolled cell growth and cancer, highlighting its role in safeguarding genomic integrity and preventing oncogenesis. By targeting PTEN miRNAs involved in the regulation of angiogenic pathways, it is possible to suppress the formation of new blood vessels within the tumor microenvironment, thereby limiting its growth and spread^96^. This approach holds great potential for developing novel therapeutic strategies aimed at disrupting the blood supply to breast tumors, ultimately contributing to improved patient outcomes^97^. Further investigation into the precise mechanisms underlying miRNA-mediated inhibition of tumor angiogenesis is warranted to translate these findings into clinical applications for the benefit of breast cancer patients.

#### microRNA Inhibiting tumor invasion and metastasis.

Dysregulation of microRNAs (miRNAs) is the hallmark of triple-negative breast cancer (TNBC), which is closely involved with its growth, metastasis, and recurrence. Creating multi-target, on-demand, non-coding RNA regulatory nanoplatforms (MTOR) marks a remarkable leap forward in accurately managing dysregulated miRNAs^98, 99^. This innovative approach has demonstrated remarkable efficacy in the suppression of triple-negative breast cancer (TNBC) growth, metastasis, and recurrence. The ability of MTOR to target multiple miRNAs and regulate their activity on-demand holds great promise for developing more effective and personalized therapeutic strategies for TNBC and potentially other types of cancer. This breakthrough offers new hope for patients and clinicians in the fight against tumor invasion and metastasis. Furthermore, miRNA-based therapeutics offer the potential for targeted and personalized treatment strategies, making them an attractive option for the management of metastatic breast cancer.

### RNA ligand-displaying extracellular vesicles for targeted delivery and treatment of breast cancer resistance

#### New technology for displaying ligands on the surface of extracellular vesicles.

Recently, a groundbreaking new technology has been developed to display ligands on the surface of extracellular vesicles^73^. This innovative method holds great promise for enhancing the targeting and delivery of extracellular vesicle-based therapeutics. Extracellular vesicles play a critical role in intercellular communication by facilitating the transfer of miRNA cargos to recipient cells^100–102^. This natural process has been observed to facilitate the spread of cancer cells and contribute to metastatic progression^103^. Readers should refer to additional review papers on this topic for more in-depth information on extracellular vesicle biogenesis, biochemical characteristics, and stimulus conditions^104–107^. Extracellular vesicles can fuse with endosome membranes via receptor-mediated endocytosis, highlighting their potential in clinical trials. Around 100 global clinical trials are investigating extracellular vesicle applications for targeting various diseases, including cancer diagnosis, identification of specific cancer types, and targeted drug therapy delivery. Extracellular vesicle protein profiling shows excellent potential in distinguishing between cancer patients at different stages and across cancer types, holding promise for improving cancer diagnosis^108, 109^. Furthermore, extracellular vesicles could be used for personalized tumor vaccination, stimulating tumor-specific immunity and potentially serving as a source of individual-specific antigens^71, 110–113^. Extracellular vesicles (EVs) can be designed to display specific cell surface ligands for precise delivery to cancer cells. Two primary methods are used to modify the membrane surface of EVs: (i) chemical attachment of molecules to EVs ex vivo or (ii) genetic engineering of cells to express desired molecules in EVs through gene transfection for specific applications^114^.

#### In vitro chemical methods for displaying ligands on the surface of extracellular vesicles.

The extracellular methods involve using RNA/DNA aptamers, chemical ligands, or short peptides as ligands displayed on the surface of extracellular vesicles. Studies have shown that many chemical substances (e.g., folate^115^) possess specificity in binding to cellular receptors. Therefore, these ligands have been used as conjugates with RNA nanoparticles to display on the surface of extracellular vesicles^72, 116–120^. The examples include specific extracellular vesicle -targeting peptides such as CD63-specific extracellular vesicle marker peptide CP05^121^, neuropilin-1-targeted peptide RGE^122^, immunomodulatory protein FasL^123^, and bone marrow stromal cell (BMSC)-specific aptamer^124^. It has been reported that chemically modified extracellular vesicles can successfully deliver therapeutic cargoes such as curcumin^122, 125^, chemotherapy drugs^126–129^, photosensitizers^130^, and quantum dot photothermal agents^131^.

#### Genetic engineering approach in cells for displaying ligands on the extracellular vesicle surface.

Gene editing is an indirect method and practical approach for modifying extracellular vesicles. Despite its challenges, it can be achieved through transfecting extracellular vesicle -producing cells with plasmid vectors to transform target molecules. Plasmid transfection of cells with ligands or portions encoding for cell membrane-bound or intracellular organelle-bound ligands. Two main classes of transmembrane proteins are commonly found on the surface of extracellular vesicles. Lysosome-associated membrane protein 2b (Lamp2b) is the most common non-specific protein compound on the surface of extracellular vesicles^114^. Alvarez et al. proved the potential of “self” extracellular vesicles for delivering therapeutic drugs and targeting moieties^132^ by demonstrating that dendritic cells expressing FLAG-Lamp2b produced extracellular vesicles carrying chemically modified siRNA^133^. The tetraspanin superfamily CD63/CD9/CD81, a group of transmembrane proteins, has been observed on the surface of extracellular vesicles as another major component^114^. Studies have reported that T7 peptide-modified extracellular vesicles and the RAGE-binding peptide of exosomal membrane-integrated protein Lamp2b exhibit higher intracellular delivery efficiency than unmodified extracellular vesicles^134, 135^. By fusing CD9 with the RNA-binding protein HuR, engineered extracellular vesicles designed for loading RNA have shown a relatively high-affinity interaction with miR-155^136^. This research indicates that engineered extracellular vesicles can effectively accumulate in recipient cells and possess a higher capacity for recognizing endogenous targets. Several alternative approaches involve the utilization of receptor membrane proteins found on specific extracellular vehicles, such as epidermal growth factor receptor (EGFR)^137^, Glycosylphosphatidylinositol (GPI)^137^, HER2^138^, platelet-derived growth factor receptor (PDGFR)^139^, and the C1C2 domain of lactadherin^125, 140^. These proteins have demonstrated heightened affinity for cancer cells and improved targeting capabilities in RNAi-mediated cancer diagnosis and treatment.

#### Exploring the Benefits and Strategies of Employing RNA Nanotechnology for Ligand Display on Extracellular vesicle Surfaces.

RNA nanotechnology possesses unique advantages and properties that make it a promising tool in nanomedicine. Its self-assembling capability enables precise customization of nanoscale devices for various medical uses. Additionally, RNA nanoparticles demonstrate excellent biocompatibility and low immunogenicity, ideal for in vivo applications. Their programmable nature allows for targeted drug delivery, imaging, and therapy. This technology links RNA-based nanodevice design and extracellular vehicles surface engineering, offering potential applications in targeted drug delivery and precision medicine, promising novel therapeutic strategies.

#### RNA nanoparticles have exhibited exceptional in vivo stability, presenting a versatile foundation for engineering nanoparticles of various sizes and configurations.

RNA nanotechnology was conceived in 1998^141^, to construct nanoscale particles primarily composed of short oligonucleotide chains, including a core framework and functional modules^50, 142, 143^. Since its discovery, the field has expanded to explore the clinical potential of constructing RNA nanoparticles. To be used in vivo, RNA nanoparticles require high thermodynamic stability to prevent dissociation under harsh conditions or during circulation in the body. For instance, the ideal Cas9:sgRNA ratio for systemic Cas9-mediated endothelial cell editing depends on the relative stability of the two molecules^144^. RNA in biological cells is typically a single-stranded molecule. The 2’-OH promotes hydrolysis of the phosphodiester bonds, leading to chemical cleavage and RNA degradation. Fortunately, RNA has the advantage over other nanocarrier platforms because its biological availability and strength can be modulated to achieve the desired functionality. The Zamore laboratory has demonstrated that PIWI-interacting RNA (piRNA) chemical lability, involved in silencing transposons^145^, depends on 3’ end trimming and 2’-O-methylation. According to their research, the complementary dependence of piRNA contributes to chemical lability by blocking 3’ end 2’-O-methylation without the need for base pairing with piRNA seed and 3’ sequences^146^. At the same time, it is true that incorporation of 2’-O-methyl RNA nucleotides at the cleavage site of siRNA by nucleases can lead to a 10-fold extension of siRNA half-life^147^. The Guo laboratory has discovered a heat-stable high melting temperature (Tm) three-way junction (3WJ) that can be used to construct dozens of highly stable RNA nanoparticles (Figure 3)^148, 149^. Additionally, the inclusion of 2’-fluoro (2’-F) or 2’-O-methyl (2’-OMe)^120^ modified nucleotides on the RNA nanoparticles results in stable and uniform shapes without aggregation, and they are less susceptible to degradation by RNases and serum^149^. Therefore, the RNA oligonucleotides used for constructing RNA nanoparticles serve as natural, versatile anionic polymers, akin to building self-assembling nanoparticles like Lego bricks.

#### RNA aptamers are efficient ligands for receptor binding.

Due to challenges in selection and preparation, high production costs, and issues with stability and cross-reactivity, monoclonal antibodies have limited capabilities as detection reagents in biotechnology^150^. RNA aptamers, consisting of short oligonucleotide sequences, exhibit the remarkable ability to target with high affinity and specificity, akin to the binding observed in antigen-antibody interactions. This characteristic makes them valuable tools in various applications, including targeted drug delivery, biosensing, and diagnostics^151, 152^. Aptamers offer numerous advantages over antibodies, including enhanced stability^153, 154^, longer shelf life^155^, smaller sizes^152, 156, 157^, cost-effectiveness, and quicker processing and generation times^158^. These attributes make aptamers a compelling choice in various applications. Researchers have utilized cell-SELEX (Systematic Evolution of Ligands by Exponential Enrichment) to identify biomarkers for cancer treatment and therapeutic applications to generate oligonucleotide aptamers. This method allows for the identification of specific nucleic acid sequences that can bind to target molecules, providing potential candidates for use in cancer therapy. By utilizing cell-SELEX, researchers can systematically evolve and enrich nucleic acid ligands, leading to the identification of promising biomarkers and therapeutic agents for the treatment of cancer^159^. Aptamers have found diverse applications in various types of cancer, including colorectal cancer^160^, breast cancer^161, 162^, osteosarcomas^163^, prostate cancer^162^, hepatocellular carcinomas^164, 165^, and colon cancer^166, 167^. Among these, the RNA aptamers targeting prostate-specific membrane antigen (PSMA) have emerged as particularly successful and effective^168, 169^. This family of aptamers has been widely utilized and is considered one of the most promising nucleic acid aptamers.

#### RNA nanoparticles have shown proficiency in transporting various components efficiently.

RNA nanoparticles, which self-assemble from multiple component chains, can precisely incorporate functional groups, allowing the creation of functional domains in their branched regions through simple sequence extensions and 5’/3’ end modifications. Modifications such as −NH2, −COOH, maleimide, −SH, alkyne, and azide facilitate the attachment of targeting ligands, imaging agents, and therapeutic molecules^170, 171^. This expands the usability of RNA nanoparticles in biomedical and biotechnology fields. They carry chemotherapeutic drugs and ligands for targeted cancer drug delivery and fluorescent dyes for live cell imaging. RNA-decorated extracellular vesicles show promise in enhancing target specificity, protecting cargo, enabling targeted delivery, improving loading, reducing toxicity, and monitoring outcomes, making them ideal for RNAi-mediated cancer treatment.

## CONCLUSION AND PERSPECTIVE

Breast cancer patients often struggle with drug resistance, compromising their treatment’s success. Existing strategies are inadequate in countering this issue. Extracellular vesicles offer potential in cancer therapy via targeted drug or siRNA delivery but face challenges in achieving target specificity. This review proposes adopting RNA nanocarriers with cell-specific ligands to enhance siRNA delivery and resolve these specificity issues. RNA nanocarriers provide benefits such as multivalency and effective tumor accumulation. Incorporating RNA nanotechnology into extracellular vesicles could advance cancer treatment. Ligand-displayed RNA nanotechnology may also enhance siRNA delivery to cytosolic compartments, mitigating endo-lysosomal entrapment. In addition, it is imperative to employ robust imaging techniques to effectively examine extracellular vesicle (EV)-mediated membrane fusion. This review highlights the potential of RNA nanotechnology to enhance extracellular vesicle biology for RNAi-based cancer therapy.

## Figures and Tables

**Table 1.** Breast cancer therapeutic drugs and their molecular mechanisms of drug resistance

Therapeutic strategy	Drugs	Molecular mechanisms of drug resistance
Chemotherapy	Anthracyclines:	
	Doxorubicin Epirubicin	drug efflux (up-regulation of ABC transporter: P-gpP-gpP-gp), drug inactivation and effluxed by MRP, non-coding RNA (lncRNA, mi-RNA, circRNA: circKDM4C down-regulating miR-548p/PBLD axis), epigenetic regulation, extracellular vesicle^177–180^
	Texans:	
	Paclitaxel Docetaxel	epigenetic regulation, non-coding RNA (lncRNA, such as DCST1-AS1, circRNA), extracellular vesicle^178, 179, 181, 182^
	5-fluorouracil (5-FU)	non-coding RNA (lncRNA)^181^
	Platinum agents:	
	Cisplatin Carboplatin	epigenetic regulation, non-coding RNA (lncRNA: SNHG1 regulates miR-381/EZH2/miR-381-MDR1 axis or DXL6 regulates miR-199b-5p/PXN), extracellular vesicle^179, 181–183^
	Gemcitabine	extracellular vesicle^182^
Hormone therapy	SERMs:	
	Tamoxifen	epigenetic regulation, extracellular vesicle, MUC-1, non-coding RNA (lncRNA, such as UCA1 regulates the EZH2/p21 axis, the PI3K/AKT pathway, inhibits miR-18a or DILA1 inhibits Cyclin D1 phosphorylation at Thr286)^179, 181, 182, 184^
Target therapy	anti-HER2 antibody:	
	Trastuzumab	PTEN loss, down-regulation or loss of HER2 expression, HER2 mutation/truncated form (p95HER2), over-activation of the alternative survival signaling pathway (PI3K/AKT/mTOR), alters HER2 receptor dimerization (HER3), antigen masking by MUC-4, CD24^−/low^/CD44+ BCSCs, epigenetic regulation, extracellular vesicle^179, 182, 185–187^
	Pertuzumab	S310F mutation of HER2 protein results in impacting Pertuzumab binding^185^
	anti-HER2 ADCs:	
	T-DM1	ROR1 and induces self-renewal of BCSCs, truncated form (p95HER2), antigen masking by MUC-4, DM1 efflux (up-regulation of ABC transporter: P-gpP-gpP-gp), lysosomal dysfunction, activation of survival signaling pathways through PI3K/AKT/mTOR axis, TME, stromal factors^188, 189^ deruxtecan efflux (up-regulation of ABC transporter: P-gpP-gpP-gp), low
	T-Dxd	SLX4 expression or mutation^189, 190^
	TKIs:	
	Lapatinib	activation of RANK signaling boosts phosphorylation of IκB and p65, miRNA-221 inhibits the expression of p27^kip1^ by targeting its 3’ UTR, constitutive activation of PI3K/AKT, MEK, and MAPK pathways^177, 191^
	Neratinib	TG2 expression activates NF-κB/STAT3 signaling loop^177^
	CDK4/6 inhibitors:	
	Palbociclib Ribociclib Abemaciclib	intrinsic: CDK4/6 overexpression, loss of Rb or ER, over-expression of p16 or CDK-2, miRNA, extrinsic: over-activation of oncogenic signaling pathways (PI3K, MAPK, and TGFBR); alternation in the Hippo pathway, HDAC over-expression, ER over-activation^191^
	Other inhibitors:	
	Everolimus (mTOR) Alpelisib (PI3K) Capivasertib (AKT)	truncated form (p95HER2), upregulation of other receptors can compensate for the inhibited HER2^185^
Immunotherapy	Pembrolizumab	epigenetic regulation, extracellular vesicle^179, 182^

**Table 2.** Natural compounds antagonists to nAChR. Summary of nAChR inhibitors from animals, plants, and algae

Origin	Extraction from	Inhibitor	Target (Species)
Animal	Scorpion venom	OSK1^192^	α7 (Human)
	Ladybird beetles	Harmonine^193^	α7 (Human)
	Snake venom	α-bungarotoksin/vurtoxin/ waglerins^194–196^	α7 (Human)
	Sea snails	Conotoxins^197^	α7, α 3β2, α3β4, α4β2, α6, α9, α10, α1β1 γδ and α1β1 δε (Human)
	Sea worm	Nereistoxin^198^	nAChRs (Mammalian)
	Sea slugs	Molleamine C^199^	nAChR (Mouse, Rat)
	Soft corals	Lophotoxin/cembranoids^200^	α4β2-AChR (Human)
	Ascidians	Lepadin B/pictamine^201^	α7, α4β2 (*Xenopus Oocytes*)
	Marine sponges	poly-APS/makaluvamine G^202^	α7 (Human)
	Ivory mollusc	Neusurugation^203^	nAcChoR (Human)
Plant	Black cohosh	Cimicifugoside^204^	nAChR (Bovine)
	chamomile	Bisabolol^205^	α7 (*Xenopus Oocytes*)
	chondrodendron	D-tubocurarine^206^	α7 (Human)
	Matis poison	Bisbenzylisoquinoline alkaloids^207^	α7 (Human)
	delphinium	Methyllycaconitine^208^	α7 (Human)
	Essential oils	Carvacrol/β-Caryophyllene^209, 210^	nAChR (Human)
	Elm-leaf grewia	Microgrewiapine A^211^	α3β4, α4β2 (Human)
	Tobacco leaves	Cembranoids (β-CBT-diol)^212^	α7, α4β2 (Human)
	Borneo camphor	Boneol^213^	nAChR (Human)
	Pu-erh tea	Pu-erh tea extract^214^	α9 (Human)
	Green tea	Epigallocatechin Gallate^215^	α9 (Human)
	Garcinia indica	Garcinol^175^	α9 (Human)
	many plants, fruits, and vegetable	Luteolin^216^	α9 (Human)
	vegetables	Quercetin^216^	α9 (Human)
Algae	Unicellular dinoflagellate algae	Pinnatoxins/Spirolides/Gymnodimines^217^	α7 (Human)

**Table 3.** Different applications and functions of siRNA and microRNA in treating breast cancer

RNA Type	Therapeutic Role	Specific Function	Example
**siRNA**	RNA-Mediated Gene Therapy	siRNA Silencing Multidrug Resistance (MDR)	To overcome MDR, self-assembled polypeptide nanoparticles LAH4-L1-siRNA (PNLS) were prepared and loaded with a siRNA (siMDR1)^75^. siRNAs targeting ABCB1 and ABCG2 transcripts resulted in a more robust increase of chemosensitivity^76^.
		siRNA Inhibiting Tumor Proliferation	siRNA specifically targeted to metallothionein-IIa (MT-IIA) messenger RNA (mRNA) and survivin^77^.
		siRNA Inhibiting Tumor Angiogenesis	ICAM-1-targeted, Lcn2 siRNA- encapsulating liposome binds human TNBC MDA-MB-231cells significantly inhibit angiogenesis^80^.
		siRNA Suppressing Tumor Invasion and Metastasis	The RNA silencing CCDC80 significantly increased sensitivity to chemotherapy agents in OXA-resistant cell lines and a mouse model^82^.
**microRNA**	Multivalent Expression Changes	microRNA Overcoming Gene-Induced Multidrug Resistance (MDR) in Chemotherapy	miRNA-765 promote multidrug resistance via targeting BATF2 in gastric cancer cells^91^.
		microRNA Overcoming Tumor Proliferation and Apoptosis	MiRNA-138-5p is a strong tumor suppressor that targets PD-L-1, reducing proliferation and inducing apoptosis in breast cancer cells^92^.
		The Role of microRNA in Inhibiting Tumor Angiogenesis	miRNAs impact phosphatase and tensin homologs (PTEN) to suppress neovascularization within the tumor microenvironment^96^.
		microRNA Inhibiting Tumor Invasion and Metastasis	miRNA target on multi-targeting and on-demand non-coding RNA (MTOR) inhibit TNBC cells invasion and metastasis^99^.

## ABBREVIATIONS:

ABC: ATP-binding cassette
P-pgpP-gpP-gp: P-glycoprotein
MRP: multidrug resistance-associated protein
KDM4C: lysine demethylase 4C
PBLD: phenazine biosynthesis-like domain-containing protein
SNHG1: small nucleolar RNA host gene 1
DCST1-AS1: DCST1 Antisense RNA 1
EZH2: enhancer of zeste 2 polycomb repressive complex 2 subunit
DXL6: distal-less homeobox 6
PXN: paxillin
SERM: selective estrogen receptor modulator
UCA1: urothelial cancer associated 1
BCSCs: breast cancer stem cells
ROR1: type 1 receptor tyrosine kinase-like orphan receptor
DM1: emtansine
Dxd: deruxtecan
MUC: mucin
TME: tumor microenvironment
SLX4: structure-specific endonuclease subunit
UTR: untranslated region
TG2: transglutaminase 2
ADC: antibody-drug conjugate
TGFBR: transforming growth factor-beta receptor
HDAC: Histone deacetylase
TME: tumor microenvironment
CSC: cancer stem cell
EMT: epithelial-mesenchymal transition
3-WJ: three-way junction
pRNA: packaging RNA
RISC: RNA-induced silencing complex
NP: nanoparticle
